# Exogenous hormones supplementation improve adventitious root formation in woody plants

**DOI:** 10.3389/fbioe.2022.1009531

**Published:** 2022-09-13

**Authors:** Yanqiu Zhao, Yinjie Chen, Cheng Jiang, Meng-Zhu Lu, Jin Zhang

**Affiliations:** ^1^ State Key Laboratory of Subtropical Silviculture, Zhejiang A&F University, Hangzhou, Zhejiang, China; ^2^ The Engineering Research Institute of Agriculture and Forestry, Ludong University, Yantai, Shandong, China

**Keywords:** exogenous hormone, auxin, adventitious root formation, woody plants, clonal propagation

## Introduction

Compared to seed propagation, clonal propagation is a simpler, faster, and more efficient method to rapidly expand millions of cuttings from elite germplasms (Gonin et al., 2019; Solgi et al., 2022). Adventitious roots (AR) formed from above-ground organs such as stems and leaves are crucial for clonal propagation, which is mainly controlled by the balance of endogenous and exogenous hormones (Lakehal and Bellini, 2019). Therefore, understanding the mechanisms of AR formation in woody species is important for large-scale vegetative propagation of economically and horticulturally important tree species.

Due to the recalcitrance of AR formation in many tree species, the application of exogenous hormones becomes a major approach for optimizing clonal propagation (Legué et al., 2014). Here, we focused on woody species and compared the selection and dosages of exogenous hormones that promote AR formation in cuttings or tissue culture. In addition, we proposed the opinion of promoting AR formation by balancing endogenous and exogenous hormones, thereby accelerating the tree breeding process.

## Adventitious root formation is controlled by endogenous hormonal balance

**FIGURE 1:**
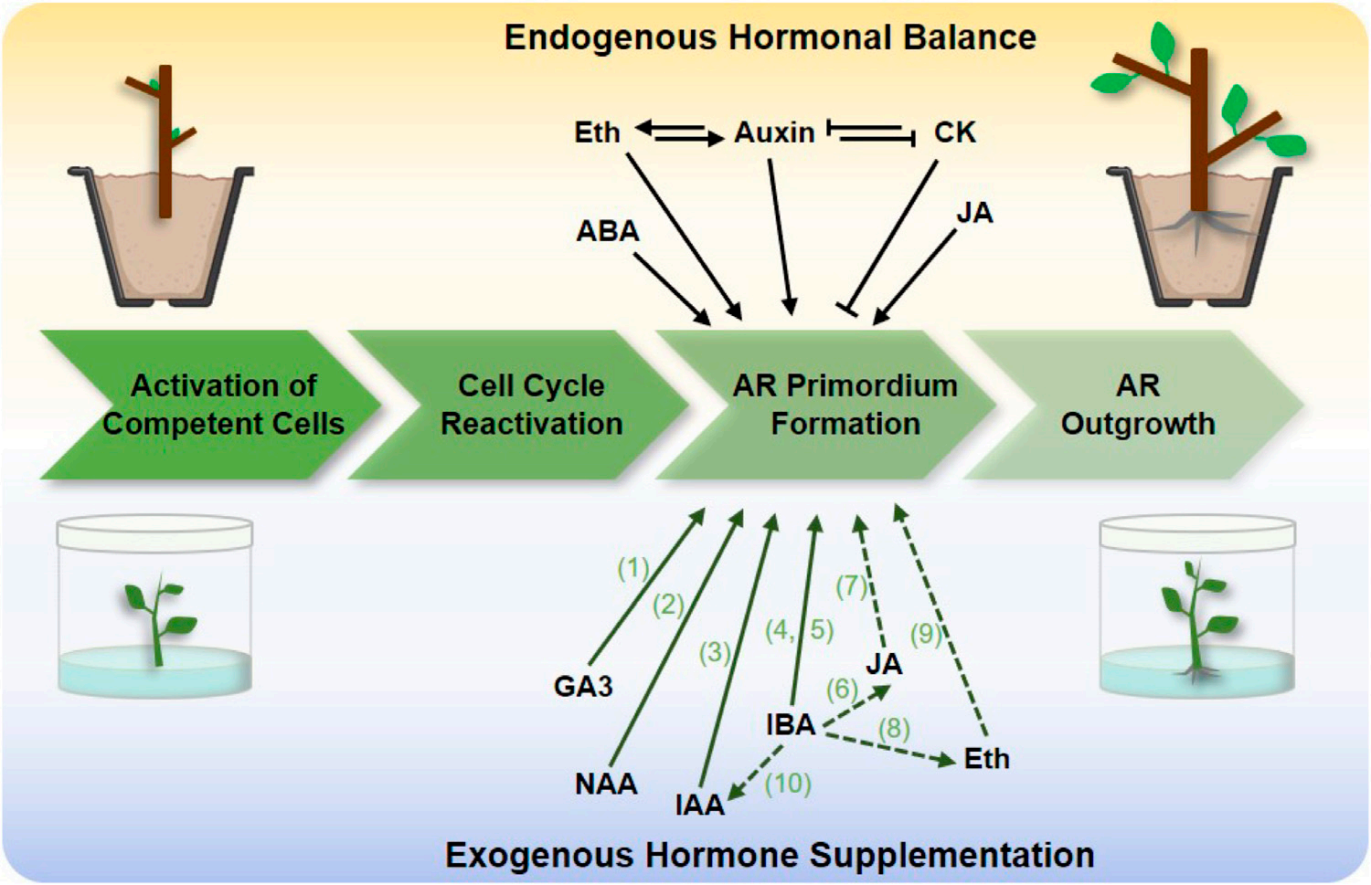
Hormones affecting adventitious root (AR) formation in clonal propagation by cuttings and tissue culture of a woody plant. The top panel is endogenous factors that directly induce AR primordium formation, the lower panel represents the effects of exogenous hormone supplementation for rooting. The phytohormones include jasmonic acid (JA), ethylene (Eth), cytokinins (CKs), abscisic acid (ABA), indole-3-butyric acid (IBA), indole acetic acid (IAA), 1-naphthalene acetic acid (NAA), gibberellin 3 (GA3). The dash lines represent the endogenous hormone response to exogenous hormone application. Green letters represent references 1–10: 1) Ford et al. (2002); 2) de Almeida et al. (2015); 3) de Almeida et al. (2015); 4) Fett-Neto et al. (2001); 5) Harfouche et al. (2007); 6) Liu et al. (2021); 7) Liu et al. (2021); 8) Bai et al. (2020); 9) Kilkenny et al. (2012); 10) Munir et al. (2021). The arrows and “T” at the end of lines represent positive and negative regulation, respectively.

Four stages are included in the AR formation process (Figure 1): activation of competent cells, cell cycle reactivation, AR primordium formation, and AR outgrowth (Legué et al., 2014). Previous studies have suggested that the formation of AR is controlled by multiple endogenous and exogenous factors (Gonin et al., 2019). Among these, phytohormones play an important role (Pacurar et al., 2014), and auxin seems to be the master regulator controlling AR formation, as it responds to rooting-competent tissue, plays a decisive role in cell fate, and activates signaling regulatory networks (Druege et al., 2016).

For changes of endogenous hormones in AR formation in woody species, multiple experimental evidences confirmed that the induction of auxin-related processes during AR formation in black walnut (*Juglans nigra* L.) (Stevens et al., 2018), black locust (*Robinia pseudoacacia* L.) (Uddin et al., 2022), and *Populus tremula* (Vaičiukynė et al., 2019), which may be related to auxin-promoted cell wall loosening and stretching (Wei et al., 2019). Furthermore, the difference between easy-to-root and difficult-to-root genotypes is attributed to changes in the concentration of inactive forms of auxin conjugates (Gonin et al., 2019). Moreover, there is a complex regulation, balance, and signaling cross-talk between auxin and other phytohormones (Figure 1). For example, ethylene (Eth) positively regulates AR formation through modulating auxin transport in tomato (Negi et al., 2010), and cytokinin interacts with Eth and auxin pathways to antagonize AR development in poplar (Ramírez-Carvajal et al., 2009). Besides that, other hormonal signaling pathways are also known to affect rooting independent of auxin. For example, abscisic acid (ABA) accumulation is found in AR formation in birch (*Betula pendula*) B1 genotype 6-fold higher than in non-rooting birch B2 genotype (Vaičiukynė et al., 2019); jasmonic acid (JA) content was accumulated after cutting injury to beneficial for rooting in *Platycladus orientalis via* activating the regeneration of stem cells (Liu et al., 2021). Therefore, it is necessary to maintain the endogenous hormone balance in rooting during the clonal propagation process.

## Exogenous hormones supplementation promotes AR formation

For perennial woody plants, stem cutting and tissue culture are the most commonly used clonal propagation techniques (Winkelmann, 2013). According to the changes in the endogenous hormone balance in rooting, the application of exogenous hormones is the most effective means to promote AR formation (Figure 1). For tissue culture propagation, rooting of microshoots was achieved *in vitro* in the presence of exogenous hormones supplementation and was also successfully grown in a rooting mixture of peat and perlite (Figure 1).

As a central player in AR formation, natural auxins and their synthetic analogs are not only the most powerful exogenous stimulators but also used empirically for rooting cuttings in different species (Legué et al., 2014). The exogenous supplementation of cuttings with the auxin analog indole-3-butyric acid (IBA) can strongly promote AR induction in woody species such as chestnuts (*Castanea*) (Vielba et al., 2020), eucalyptus (*Eucalyptus globulus*) (Fett-Neto et al., 2001; Vilasboa et al., 2019), and teak (*Tectona grandis*) (Azamal and Sayyada, 2012) (Table 1). In *C. sativa*, endogenous IAA content increased with IBA treatment (Vielba et al., 2020), suggesting that IBA treatment affects AR development by modulating endogenous auxin level. In addition, GA_3_ pre-treatment of cherry (*Prunus avium*) stock resulted in the AR formation of cuttings increased (Ford et al., 2002). Therefore, the application of appropriate exogenous hormones has a significant effect on the rooting of cuttings of different species.

**TABLE 1:** Exogenous hormone application of different woody species during clonal propagation.

Species	Clonal propagation methods	IBA (mg/L)	NAA (mg/L)	IAA (mg/L)	GA3 (mg/L)	Reference
*Castanea sativa*	Tissue culture	2,000	—	—	—	Vielba et al. (2020)
*Castanea dentata*	Tissue culture	9,000	—	—	—	Vielba et al. (2020)
*Castanea sativa* × *Castanea crenata*	Tissue culture	5,000	—	—	—	Vielba et al. (2020)
*Malus domestica*	Tissue culture	0.6	—	—	—	Bai et al. (2020)
*Robinia pseudoacacia* L.	Tissue culture	0.6	—	—	—	Uddin et al. (2022)
*Cedrela fissilis* Vellozo	Tissue culture	10	—	—	—	Ribeiro et al. (2022)
*Azadirachta indica*	Tissue culture	1	—	—	—	Quraishi et al. (2004)
*Santalum album*	Tissue culture	50	—	—	—	Bhargava et al. (2018)
*Anacardium occidentale* L.	Tissue culture	0.1	—	—	—	Camille et al. (2021)
*Eucalyptus globulus*	Tissue culture	10	—	—	—	Fett-Neto et al. (2001)
*Malus × domestica* Borkh.	Tissue culture	3,000	—	—	—	Li K et al. (2021)
*Vitis* sp.	Tissue culture	0.1	—	—	—	Chang et al. (2022)
*Robinia pseudoacacia*-148	Tissue culture	—	0.3	—	—	Munir et al. (2021)
*Populus* × *euramericana* ‘Neva'	Tissue culture	0.3	0.5	—	—	Liu et al. (2016)
*Populus alba* × *P*. *glandulosa*	Tissue culture	0.05	0.02	—	—	Wen et al. (2022)
*Eucalyptus grandis*	Tissue culture	—	—	10	—	de Almeida et al. (2015)
*Prunus avium*	Tissue culture	—	—	—	10	Ford et al. (2002)
*Populus alba*	Cutting	6,000	—	—	—	Harfouche et al. (2007)
*Pinus banksiana*	Cutting	—	1,000	—	—	Browne et al. (1997)
*Malus xiaojinensis*	Cutting	3,000	—	—	—	Li X et al. (2021)
*Prunus avium*	Cutting	1,250	—	—	—	Ford et al. (2002)
*Olea europaea* ‘Manzanilla'	Hadwood cutting	3,000	—	—	—	Khajehpour et al. (2014)

## Application of exogenous auxin in cutting and tissue culture propagation of woody species

Cutting and tissue culture offer prospects for faster multiplication of elite trees (Winkelmann, 2013). Unfortunately, many species, such as cashew (*Anacardium occidentale* L*.*) and chestnut, are highly recalcitrant to *in vitro* culture and clonal propagation as the astrict to form AR (Camille et al., 2021). In order to overcome the difficulty in rooting, the basal part of cuttings can usually be treated with high concentrations of auxin for root production and then plant for cutting regeneration, or the stem segment explants are cultured in Murashige and Skoog (MS) medium supplemented with various concentrations of auxin or its analogs, such as indole acetic acid (IAA), IBA, and 1-naphthalene acetic acid (NAA) (Winkelmann, 2013). Transcriptomic profiles of NAA- or IBA-induced AR formation indicated that exogenous NAA and IBA stimulated multiple pathways including phytohormone signal transduction and metabolic pathways to promote rooting in black locust tissue culture (Munir et al., 2021) and nodal cuttings of tea (*Camellia sinensis* L.) (Wei et al., 2014), respectively. This suggests that the promotion of AR by exogenous auxin may involve other hormones or metabolic signals.

In cutting, the dipping method of auxin application is commonly used to induce AR formation, which means a short pulse of a high concentration of the hormone enters the plant tissues through the cut surface of cuttings and will eventually enter the cells by pH trapping or the activity of influx carrier proteins. Shoot cuttings of poplar grown *in vitro* in the presence of IAA inhibitors caused complete inhibition of rooting (Bellamine et al., 1998). In contrast, IBA- or NAA-treatment induced root primordia in white poplar (*Populus alba*) cuttings and increased the number of AR per cutting (Harfouche et al., 2007). Moreover, Browne et al. (1997) compared the rooting frequency of jack pine (*Pinus banksiana*) cuttings of different tree ages (3, 7, and 12 years) and found that NAA treatment generally increased rooting by 2-3 fold compared to controls. In apple (*Malus domestica* Borkh.) propagation, the rooting rate in cutting is significantly higher after treatment under 3 g/L IBA with 50 mM H_2_O_2_ since H_2_O_2_ significantly enhances the effect of IBA on rooting (Xiao et al., 2014), or only with 3 g/L IBA (Li X et al., 2021) for 1 min and then inserted into fine sand and incubated in a mist solar greenhouse, and study has demonstrated that IBA stimulates the production of Eth to stimulate AR formation in apple rootstock propagation (Bai et al., 2020; Devi et al., 2021). Olive is one of the hard-to-rooting plants, the hardwood cuttings of olive (*Olea europaea* ‘Manzanilla') treated under 3 g/L IBA showed a 1.8-fold increase in rooting rate (Khajehpour et al., 2014). The basal of cherry cuttings dip in 1.25 g/L IBA and then plant in pots containing peat significantly increases the number of AR (Ford et al., 2002). The studies described indicate that IBA and NAA as commonly used exogenous auxin in the propagation of forest tree cuttings promote AR formation by the dipping method.

During the root regeneration from tree microshoots, exogenous application of NAA or IBA promotes rooting mainly in two ways: prolonged incubation with low concentrations in the medium or transient dipping. For the dipping method, the effect of pulse treatment of 50 mg/L IBA on *in vitro* propagated shoots for 48 h and then transferred to MS medium provided a maximum percentage of root induction for sandalwood (*Santalum album*) (Bhargava et al., 2018). Cuttings were treated with concentrations of 9 g/L IBA for 10 s and then transferred to MS medium for AR inducing in chestnut propagation. Moreover, effective root regeneration of eucalyptus microshoots is obtained by a 4-days exposure to 10 mg/L IBA during the AR induction step (Fett-Neto et al., 2001). The previous study shows that plantlets obtained by culturing in media with exogenous adjuvants are healthier compared to transient immersion (Vielba et al., 2019). Therefore, all of the shoots of neem (*Azadirachta indica*) formed AR under MS medium with 1 mg/L IBA supplementation (Quraishi et al., 2004), and the highest percentage of AR formation was cultured with 0.1 mg/L IBA in the medium of cashew propagation (Camille et al., 2021). Exogenous application of 0.3 mg/L NAA into MS medium stimulated AR formation in black locust propagation (Munir et al., 2021). Based on the above, the application of exogenous IBA or NAA is essential for *ex vitro* rooting of tree species in which cuttings or tissue culture is the main propagation method.

Poplar is not only an important economic crop but also a woody model species. It comprises about 30 species with huge varieties in rooting ability, which is related to their sensitivity to exogenous hormones (Luo et al., 2021). Although several poplar species are easy-to-root, exogenous hormone application can accelerate root initiation and promote root quality in hybrid poplars. For example, 0.05 mg/L IBA with 0.02 mg/L NAA is used to promote root initiation in hybrid poplar ‘84K' (*P. alba* × *P. glandulosa*) (Wen et al., 2022), while 0.3 mg/L IBA with 0.5 mg/L NAA are required in hybrid poplar (*Populus* × *euramericana* ‘Neva') rooting induction medium (Liu et al., 2016). In addition, exogenous IAA impacts differently on gene expression modifications in cuttings of different species of *Eucalyptus* easy- and difficult-to-root, e.g., IAA improves root number and length in *E. grandis* while with no significant effect on these parameters being observed in *E. globulus* (de Almeida et al., 2015). Together, trees with interspecific variations require different kinds and appropriate contents of auxin to promote AR formation in clonally propagated species.

## Dose-effect relationships of exogenous auxins in AR formation

The proper concentrations of plant growth regulators are important because excessive auxin concentrations may inhibit AR initiation (Pant et al., 2023), suggesting a dose-effect of exogenous hormones on AR formation. For instance, 1 mg/L IBA has a less promoting effect on AR formation than 0.1 mg/L IBA with grape (*Vitis* sp.) cuttings (Chang et al., 2022). In addition, 0.1 mg/L and 10 mg/L NAA promote and inhibit AR formation in apple cuttings, respectively (Li K et al., 2021). Conversely, increasing auxin levels correlated positively with rooting success using ‘M116' apple clonal rootstock (Patial et al., 2021), and *Eucalyptus* (*Eucalyptus pellita* × *E. grandis*) cuttings formed significantly more adventitious roots when the cuttings dipped with 8 g/L IBA than that formed in 3 g/L IBA (Kilkenny et al., 2012). Therefore, mastering the optimal concentration of IBA or NAA application in the clonal propagation of diverse woody plants can improve the rooting rate and achieve efficient *in vitro* multiplication.

## Future perspective

Adventitious rooting is indispensable for the vegetative propagation of forestry and horticultural plants. A good rooting system is necessary for plants to adapt to various environments and increase yields, as it can absorb more nutrients for the growth of the above-ground parts. Therefore, advances in the knowledge of AR formation will pave the way for optimizing clonal propagation in woody species. Internal and external factors impact AR formation, of which, auxin as the master hormone regulator seems to be the most important and central one. Despite advances in exogenous hormone selection and concentration over the past few decades, the response of inter-species variation in woody plants to different hormones and their impact on AR formation remain unclear. Therefore, the mechanism of auxin and other hormones in AR formation in diverse woody plants still needs to be analyzed, in order to improve the efficiency of clonal propagation by applying exogenous hormones.

## References

[B1] Azamal H. Sayyada K. (2012). Role of anthraquinones as a marker of juvenility and maturity in response to adventitious rooting of *Tectona grandis* . Am. J. Plant Physiology 7, 220–231. 10.3923/ajpp.2012.220.231

[B2] Bai T. Dong Z. Zheng X. Song S. Jiao J. Wang M. (2020). Auxin and its interaction with ethylene control adventitious root formation and development in Apple rootstock. Front. Plant Sci. 11, 574881. 10.3389/fpls.2020.574881 33178245 PMC7593273

[B3] Bellamine J. Penel C. Greppin H. Gaspar T. (1998). Confirmation of the role of auxin and calcium in the late phases of adventitious root formation. Plant Growth Regul. 26, 191–194. 10.1023/A:1006182801823

[B4] Bhargava P. Ravindra N. Singh G. (2018). A modified and improved protocol development for *in vitro* clonal propagation of *Santalum album* L. from internodal explants. Trop. Plant Res. 5, 193–199. 10.22271/tpr.2018.v5.i2.024

[B5] Browne R. Davidson C. Steeves T. Dunstan D. (1997). Effects of ortet age on adventitious rooting of jack pine (*Pinus banksiana*) long-shoot cuttings. Can. J. For. Res. 27, 91–96. 10.1139/x96-160

[B6] Camille K. Laurent K. K. Martine B. M. Irié Z. B. (2021). Clonal propagation of cashew (*Anacardium occidentale* L.) by stem cuttings and *in vitro* adventitious shoots and roots formation. J. Animal Plant Sci. 49, 8845–8855. 10.35759/JAnmPlSci.v49-2

[B7] Chang X. Zhang K. Yuan Y. Ni P. Ma J. Liu H. (2022). A simple, rapid, and quantifiable system for studying adventitious root formation in Grapevine. Plant Growth Regul., 1–10. 10.21203/rs.3.rs-1318845/v1 35892116

[B8] de Almeida M. R. de Bastiani D. Gaeta M. L. de Araújo Mariath J. E. de Costa F. Retallick J. (2015). Comparative transcriptional analysis provides new insights into the molecular basis of adventitious rooting recalcitrance in *Eucalyptus* . Plant Sci. 239, 155–165. 10.1016/j.plantsci.2015.07.022 26398800

[B9] Devi J. Kumar R. Singh K. Gehlot A. Bhushan S. Kumar S. (2021). *In vitro* adventitious roots: a non-disruptive technology for the production of phytoconstituents on the industrial scale. Crit. Rev. Biotechnol. 41, 564–579. 10.1080/07388551.2020.1869690 33586555

[B10] Druege U. Franken P. Hajirezaei M. R. (2016). Plant hormone homeostasis, signaling, and function during adventitious root formation in cuttings. Front. Plant Sci. 7, 381. 10.3389/fpls.2016.00381 27064322 PMC4814496

[B11] Fett-Neto A. G. Fett J. P. Veira Goulart L. W. Pasquali G. Termignoni R. R. Ferreira A. G. (2001). Distinct effects of auxin and light on adventitious root development in *Eucalyptus saligna* and *Eucalyptus globulus* . Tree Physiol. 21, 457–464. 10.1093/treephys/21.7.457 11340046

[B12] Ford Y.-Y. Taylor J. Blake P. Marks T. (2002). Gibberellin A3 stimulates adventitious rooting of cuttings from cherry (*Prunus avium*). Plant Growth Regul. 37, 127–133. 10.1023/A:1020584627919

[B13] Gonin M. Bergougnoux V. Nguyen T. D. Gantet P. Champion A. (2019). What makes adventitious roots? Plants (Basel) 8, 240. 10.3390/plants8070240 PMC6681363 31336687

[B14] Harfouche A. Baoune N. Merazga H. (2007). Main and interaction effects of factors on softwood cutting of white poplar (*Populus alba* L.). Silvae Genet. 56, 287–294. 10.1515/sg-2007-0041

[B15] Khajehpour G. Jam'eizadeh V. Khajehpour N. (2014). Effect of different concentrations of IBA (indulebutyric acid) hormone and cutting season on the rooting of the cuttings of olive (*Olea europaea* Var Manzanilla). Int. J. Adv. Biol. Biomed. Res. 2 (12), 2920–2924. 10.17660/actahortic.2011.924.12

[B16] Kilkenny A. J. Wallace H. M. Walton D. A. Adkins M. F. Trueman S. J. (2012). Improved root formation in eucalypt cuttings following combined auxin and anti-ethylene treatments. J. Plant Sci. 7, 138–153. 10.3923/jps.2012.138.153

[B17] Lakehal A. Bellini C. (2019). Control of adventitious root formation: insights into synergistic and antagonistic hormonal interactions. Physiol. Plant. 165 (1), 90–100. 10.1111/ppl.12823 30159890

[B18] Legué V. Rigal A. Bhalerao R. P. (2014). Adventitious root formation in tree species: involvement of transcription factors. Physiol. Plant. 151, 192–198. 10.1111/ppl.12197 24666319

[B19] Li K. Wei Y.-H. Wang R.-H. Mao J.-P. Tian H.-Y. Chen S.-Y. (2021). Mdm-MIR393b-mediated adventitious root formation by targeted regulation of MdTIR1A expression and weakened sensitivity to auxin in apple rootstock. Plant Sci. 308, 110909. 10.1016/j.plantsci.2021.110909 34034866

[B20] Li X. Shen F. Xu X. Zheng Q. Wang Y. Wu T. (2021). An HD-ZIP transcription factor, MxHB13, integrates auxin-regulated and juvenility-determined control of adventitious rooting in *Malus xiaojinensis* . Plant J. 107, 1663–1680. 10.1111/tpj.15406 34218490

[B21] Liu D. Zhang J. Dong Y. Zhang X. Yang M. Gao B. (2016). Genetic transformation and expression of Cry1Ac–Cry3A–NTHK1 genes in *Populus* × *euramericana* ‘neva'. Acta Physiol. Plant. 38, 177–211. 10.1007/s11738-016-2195-6

[B22] Liu G. Zhao J. Liao T. Wang Y. Guo L. Yao Y. (2021). Histological dissection of cutting-inducible adventitious rooting in *Platycladus orientalis* reveals developmental endogenous hormonal homeostasis. Indu. Crop. Prod. 170, 113817. 10.1016/j.indcrop.2021.113817

[B23] Luo J. Nvsvrot T. Wang N. (2021). Comparative transcriptomic analysis uncovers conserved pathways involved in adventitious root formation in poplar. Physiol. Mol. Biol. Plants 27, 1903–1918. 10.1007/s12298-021-01054-7 34629770 PMC8484428

[B24] Munir M. Z. Ud Din S. Imran M. Zhang Z. Pervaiz T. Han C. (2021). Transcriptomic and anatomic profiling reveal etiolation promotes adventitious rooting by exogenous application of 1-Naphthalene Acetic Acid in *Robinia pseudoacacia* L. Forests 12, 789. 10.3390/f12060789

[B25] Negi S. Sukumar P. Liu X. Cohen J. D. Muday G. K. (2010). Genetic dissection of the role of ethylene in regulating auxin-dependent lateral and adventitious root formation in tomato. Plant J. 61 (1), 3–15. 10.1111/j.1365-313X.2009.04027.x 19793078

[B26] Pacurar D. I. Perrone I. Bellini C. (2014). Auxin is a central player in the hormone cross-talks that control adventitious rooting. Physiol. Plant. 151, 83–96. 10.1111/ppl.12171 24547793

[B27] Pant M. Bhandari A. Husen A. (2023). “Adventitious root formation and clonal propagation of forest-based tree species,” in Environmental, physiological and chemical controls of adventitious rooting in cuttings (Cambridge: Academic Press), 471–490.

[B28] Patial S. Chandel J. Sharma N. Verma P. (2021). Influence of auxin on rooting in hardwood cuttings of apple (*Malus* × *domestica Borkh.*) clonal rootstock ‘M 116' under malus domestica mist chamber conditions. Indian J. Ecol. 48, 429–433.

[B29] Quraishi A. Koche V. Sharma P. Mishra S. (2004). *In vitro* clonal propagation of neem (*Azadirachta indica*). Plant Cell, Tissue Organ Cult. 78, 281–284. 10.1023/B:TICU.0000025647.58548.3d

[B30] Ramírez-Carvajal G. A. Morse A. M. Dervinis C. Davis J. M. (2009). The cytokinin type-B response regulator PtRR13 is a negative regulator of adventitious root development in *Populus* . Plant Physiol. 150 (2), 759–771. 10.1104/pp.109.137505 19395410 PMC2689991

[B43] Ribeiro Y. R. D. S. Aragão V. P. M. Sousa K. R. D. Macedo A. F. Floh E. I. S. Silveira V. (2022). Involvement of differentially accumulated proteins and endogenous auxin in adventitious root formation in micropropagated shoot cuttings of Cedrela fissilis Vellozo *(Meliaceae)* . Plant Cell, Tissue Organ Cult. 148 (1), 119–135. 10.1007/s11240-021-02171-7

[B31] Solgi M. Taghizadeh M. Bagheri H. (2022). Response of black mulberry onto white mulberry rootstock to stenting (cutting-grafting) techniques and IBA concentrations. Ornam. Hortic. 28, 78–84. 10.1590/2447-536X.v28i1.2413

[B32] Stevens M. E. Woeste K. E. Pijut P. M. (2018). Localized gene expression changes during adventitious root formation in black walnut (*Juglans nigra* L.). Tree Physiol. 38, 877–894. 10.1093/treephys/tpx175 29378021

[B33] Uddin S. Munir M. Z. Gull S. Khan A. H. Khan A. Khan D. (2022). Transcriptome profiling reveals role of microRNAs and their targeted genes during adventitious root formation in dark-pretreated micro-shoot cuttings of tetraploid *Robinia pseudoacacia* L. Genes (Basel) 13, 441. 10.3390/genes13030441 35327995 PMC8950900

[B34] Vaičiukynė M. Žiauka J. Žūkienė R. Vertelkaitė L. Kuusienė S. (2019). Abscisic acid promotes root system development in birch tissue culture: a comparison to aspen culture and conventional rooting-related growth regulators. Physiol. Plant. 165, 114–122. 10.1111/ppl.12860 30367696

[B35] Vielba J. M. Vidal N. José M. C. S. Rico S. Sánchez C. (2020). Recent advances in adventitious root formation in chestnut. Plants (Basel) 9, 1543. 10.3390/plants9111543 PMC7698097 33187282

[B36] Vielba J. M. Vidal N. Ricci A. Castro R. Sanchez C. Carmen San-Jose M. (2019). Effects of auxin and urea derivatives on adventitious rooting in chestnut and oak microshoots. Isr. J. Plant Sci. 67, 52–68. 10.1163/22238980-20191113

[B37] Vilasboa J. Da Costa C. T. Fett-Neto A. G. (2019). Rooting of eucalypt cuttings as a problem-solving oriented model in plant biology. Prog. Biophysics Mol. Biol. 146, 85–97. 10.1016/j.pbiomolbio.2018.12.007 30557533

[B38] Wei K. Ruan L. Wang L. Cheng H. (2019). Auxin-induced adventitious root formation in nodal cuttings of *Camellia sinensis* . Int. J. Mol. Sci. 20, 4817. 10.3390/ijms20194817 PMC6801801 31569758

[B39] Wei K. Wang L.-Y. Wu L.-Y. Zhang C.-C. Li H.-L. Tan L.-Q. (2014). Transcriptome analysis of indole-3-butyric acid-induced adventitious root formation in nodal cuttings of *Camellia sinensis* (L.). PLoS One 9, e107201. 10.1371/journal.pone.0107201 25216187 PMC4162609

[B40] Wen S. S. Ge X. L. Wang R. Yang H. F. Bai Y. E. Guo Y. H. (2022). An efficient agrobacterium-mediated transformation method for hybrid poplar 84K (*Populus alba* × *P. glandulosa*) using calli as explants. Int. J. Mol. Sci. 23, 2216. 10.3390/ijms23042216 35216331 PMC8879841

[B41] Winkelmann T. (2013). Recent advances in propagation of woody plants. Acta Hortic. 990, 375–381. 10.17660/actahortic.2013.990.47

[B42] Xiao Z. Ji N. Zhang X. Zhang Y. Wang Y. Wu T. (2014). The lose of juvenility elicits adventitious rooting recalcitrance in apple rootstocks. Plant Cell Tissue Organ Cult. 119, 51–63. 10.1007/s11240-014-0513-5

